# Day-to-Day Variability in Meal Timing and Its Association with Body Mass Index: A Study Using Data from a Japanese Food-Logging Mobile Application

**DOI:** 10.3390/nu17223504

**Published:** 2025-11-09

**Authors:** Noriko Sato, Hiiro Terasaki, Yu Tahara, Mikiko Michie, Ariko Umezawa, Shigenobu Shibata

**Affiliations:** 1 Department of Nutritional Science, Faculty of Food and Nutritional Sciences, Japan Women’s University, Tokyo 112-8681, Japan; umezawaa@fc.jwu.ac.jp; 2 Division of Food and Nutrition, Graduate School of Human Sciences and Design, Japan Women’s University, 2-8-1 Mejiro-dai, Bunkyo-ku, Tokyo 112-8681, Japan; terasaki.hiiro@gmail.com; 3 Graduate School of Biomedical and Health Sciences, Hiroshima University, Kasumi, Minami-ku, Hiroshima City 734-0037, Hiroshima, Japan; yutahara@hiroshima-u.ac.jp (Y.T.); shibatas@hiroshima-u.ac.jp (S.S.); 4 Asken Inc., Tokyo 163-1442, Japan; michie@asken.inc; 5 Faculty of Home Economics, Aikoku Gakuen Junior College, Nishikoiwa, Edogawa-ku, Tokyo 133-0057, Japan

**Keywords:** mealtime irregularity, chronotype, age groups, body mass index

## Abstract

**Background/Objectives**: Discrepancies in mealtimes between weekdays and weekends—often referred to as “eating jetlag”—have been linked to a higher body mass index (BMI). However, in modern societies characterized by diverse work patterns, misalignment between mealtimes and the internal circadian rhythm may result not only from weekday–weekend differences but also from day-to-day variability. The aim of this study was to quantitatively assess and visualize daily mealtime variability over a 1-month period using food log data and to investigate the association between breakfast time irregularity and BMI. **Methods**: We conducted a retrospective, cross-sectional analysis using food log data (*n* = 1072; 742 women and 360 men) extracted from a popular Japanese food-logging mobile application. Mealtime irregularity was quantified using composite phase deviation (CPD). Data were stratified by sex and age tertile. **Results**: Approximately 18% of participants (women and men) exhibited irregular breakfast timing (CPD > 1 h). Multivariate regression analysis revealed that an evening-type chronotype was primarily associated with BMI among younger women, whereas breakfast time irregularity was associated with BMI among older women. **Conclusions**: Our findings suggest that daily mealtime variability is an additional chrono-nutritional factor associated with BMI. Furthermore, the chrono-nutritional factors most strongly associated with BMI may differ by age.

## 1. Introduction

Mealtime regularity, alongside the timing of meals relative to the internal circadian clock, is increasingly recognized as an important determinant of health. Irregular eating patterns—such as eating jetlag, skipping breakfast, late-night eating, and grazing—can disrupt synchronization between central and peripheral clocks or shorten daily fasting periods, thereby undermining the healthy regulation of metabolic systems [1,2]. Among these, eating jetlag, defined as the discrepancy in mealtimes between weekdays (workdays) and weekends (free days), has attracted attention due to its similarity to social jetlag (SJL; discrepancy in sleep schedules). Notably, eating jetlag affects body mass index (BMI) and cardiometabolic function [3,4,5,6].

A study by Makarem et al. involving 115 women (mean age 33 years) found that eating jetlag at the timing of the first meal was associated with a higher BMI. Furthermore, greater variability in first-meal timing—measured as the standard deviation over six to seven consecutive days of dietary data—was associated with BMI and an increase in hemoglobin a1c (HbA1c) after 1 year [6]. Their findings suggested that maintaining consistent mealtimes, particularly for earlier meals, may be beneficial for health. Analyzing day-to-day variability in breakfast timing is of particular interest, as regular morning energy intake may be essential in regulating hunger and glucose metabolism [7]. However, this aspect has not been thoroughly investigated, partly due to the difficulty of collecting long-term food log data (e.g., over 1 month), which is necessary to analyze habitual mealtime regularity, as opposed to short-term data from only a few days.

In this study, we utilized 1 month of daily food log data from a mobile health application (*n* = 1072) to characterize and visualize daily meal regularity. We aimed to quantify day-to-day mealtime irregularity using composite phase deviation (CPD) and examine its relationship with sleep–wake rhythms, mealtime, energy intake variability, and BMI. Although skipping breakfast has been associated with obesity [8,9], few participants who logged their meals daily skipped breakfast. Accordingly, we investigated whether irregular breakfast timing was associated with BMI, even among individuals who consistently ate breakfast. Analyses were stratified by sex and age to clarify the implications of mealtime irregularity within each subgroup.

## 2. Materials and Methods

### 2.1. Study Design

This retrospective, cross-sectional analysis was conducted using dietary intake data extracted from asken, a popular Japanese food-logging mobile health application with over 12 million users, as of May 2025 [10]. The asken app is widely used across different age groups, and multiple studies have confirmed the reliability of its self-reported food log data for assessing dietary habits [11,12,13,14,15]. This study utilized baseline data from the “Exercise Intervention Study Using the Mobile Health Application asken.” The study was approved by the Ethics Review Committee on Research with Human Subjects at Waseda University (No. 2020-046) and conducted in accordance with the Declaration of Helsinki. Informed consent was obtained from all participants.

### 2.2. Participants

**Figure 1:**
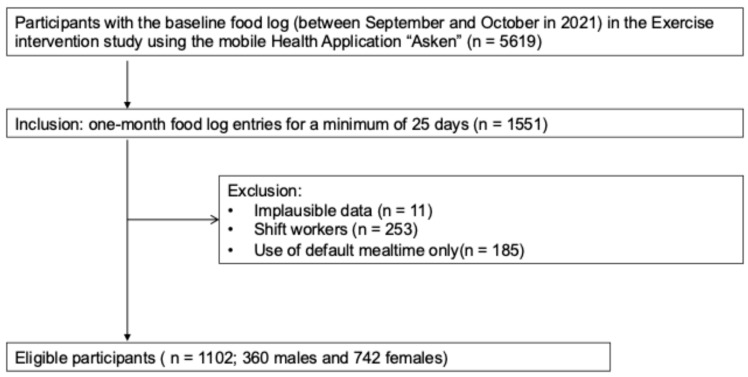
Flowchart of participant selection in the study.

A total of 5619 participants had food log data at baseline (September–October 2021). To quantify habitual meal irregularity, long-term continuous dietary records were required, so we selected participants who logged meals for at least 25 days per month (*n* = 1551). We excluded implausible data (e.g., unrealistically high nutrient intake values, impossible meal timing sequences, or meal intervals shorter than 15 min; *n* = 11), shift workers (*n* = 253), and participants who used only default mealtimes (*n* = 185). The final sample included 1102 participants (360 men and 742 women) (Figure 1).

### 2.3. Data Collection

Asken users logged their meals by recording mealtime and selecting food items and portion sizes from the in-app database or by uploading meal photos or barcode data for commercially available foods. In this study, a meal was defined as an intake of at least 50 kcal (210 kJ). The app categorizes meals as “breakfast,” “lunch,” “dinner,” or “snack,” and users select one of these categories when recording their intake. Since most Japanese adults regularly consume three meals per day, breakfast is generally the first meal of the day [16]. Given that the asken app allows mealtime entry only for the three main meals (breakfast, lunch, and dinner) and not for “snacks,” this study analyzed mealtime irregularity for the three main meals, as described in Section 2.4.

The nutritional value of each meal, including energy intake, was calculated using the 2020 Standard Tables of Food Composition in Japan (eighth edition) [17]. During the continuous recording period (minimum of 25 days), meals containing less than 50 kcal were considered “skipped meals.” Total daily energy intake was calculated as the sum of energy intake from breakfast, lunch, dinner, and snacks. The mean daily energy intake was determined by dividing the total energy intake on days without skipped meals by the number of days meals were consumed during the study period. The coefficient of variation (CV) for energy intake was calculated as the standard deviation divided by the mean energy intake.

Data on age, sex, height, weight, shift work status, and lifestyle factors were collected through an online self-administered questionnaire. Sleep habits were assessed using the short version of the Munich Chronotype Questionnaire [18]. Based on sleep onset times and wake-up times on both workdays and free days, the midpoint of sleep on free days (MSF), mean sleep duration (SD), sleep duration on free days (SD_week_), and the sleep-corrected midpoint on free days (MSFsc) were calculated [19]. Participants were classified into morning (MSFsc (h) < 2.57, e.g., MSFsc (hh:mm) < 2:34), intermediate (2.57 ≤ MSFsc (h) < 3.75), and evening (3.75 ≤ MSFsc (h), e.g., MSFsc (hh:mm) ≥ 3:45) chronotypes. Chronotype was also coded categorically as follows: 0 = morning type; 1 = intermediate type; and 2 = evening type. SJL was defined as the difference between the midpoints of sleep on workdays and free days. Physical activity was assessed using the short version of the Physical Activity Questionnaire [20] and expressed in Metabolic Equivalent of Task (MET)-hours per week.

### 2.4. Day-to-Day Irregularity in Mealtime

Day-to-day mealtime irregularity was assessed using the CPD metric as described by McHill et al. [21]. The CPD method was originally developed to quantify variability in sleep–wake timing across consecutive days to evaluate circadian disruption or misalignment [22]. For each day *i*, the CPD score was calculated based on the difference in mealtime from the previous day (day-to-day stability, $∆{DD}_{i}$) and the deviation from the mean mealtime (alignment, $∆{AT}_{i}$), as follows:

$$∆{DD}_{i}={Mealtime}_{i-1}-{Mealtime}_{i}$$

$$∆{AT}_{i}=Mean\;Mealtime-{Mealtime}_{i}$$

$${CPD}_{i}=\sqrt{{∆DD}_{i}^{2}+{∆AT}_{i}^{2}}$$

$$CPD=\frac{1}{N}\sum_{i=1}^{N}{CPD}_{i}$$

Here, *i* denotes a given day, and *N* denotes the total number of days. Given that missing data were extremely rare during the 1-month period, days with missing values were treated as consecutive to the preceding and following days.

### 2.5. Visualization and Statistical Analysis

R software (version 4.5.1) was used for data visualization and statistical analysis. Visualizations were created using the ggplot2 package to illustrate individual meal patterns, including line graphs of daily mealtimes and proportional symbol plots showing energy intake according to mealtime for the three main meals.

Spearman’s rank correlation coefficients were used to examine correlations among breakfast, lunch, and dinner CPD values.

Analyses were stratified by sex and age, as chronotypes tend to shift toward morning types with increasing age under social and environmental influences [23,24]. Participants were divided into tertiles based on age and analyzed separately for each group. For women, the tertiles were defined as T1: age < 36; T2: 36 ≤ age < 47; and T3: age ≥ 47. For men, they were defined as T1: age < 42; T2: 42 ≤ age < 53; and T3: age ≥ 53. As age-based tertile divisions depend on the study population, a sensitivity analysis was conducted among women using an alternative age stratification: Q1: age < 35; Q2: 35 ≤ age < 50; and Q3: age ≥ 50. Participants were divided into three groups based on their CPD values to examine the relationship between mealtime irregularity and other variables. The groups were defined as follows: regular (CPD < 0.5 h), slightly irregular (0.5 ≤ CPD < 1 h), and irregular (CPD ≥ 1 h). Mealtime irregularity was coded as 0 (regular), 1 (slightly irregular), and 2 (irregular).

Trends in target variables (e.g., lifestyle rhythms and energy intake variability) associated with increasing mealtime irregularity were evaluated using the Jonckheere–Terpstra test.

A multivariate linear regression model was applied to assess the association between mealtime irregularity and BMI. Covariates, including age, physical activity, and total energy intake, were standardized using Z-score transformation.

Statistical significance was set at *p* < 0.05.

## 3. Results

### 3.1. Participant Characteristics

The participants in this study were limited to consistent asken users who recorded meals nearly every day. Table 1 presents the basic characteristics of the participants. Their ages ranged from approximately 20 to 60 years, with the largest proportion in their 40s.

Given that consistent dietary logging may be associated with greater health awareness, we compared the characteristics of our participants with those from a national survey where possible. The mean BMI of participants was generally comparable to national averages across most age groups, although it was slightly higher among individuals younger than 40 years (Appendix A, Table A1). In contrast, except for women aged 60 years and older, the prevalence of breakfast skipping was substantially lower in all age groups compared with national data (Appendix A, Table A2). The time interval between breakfast and dinner was approximately 12 h in both this study and the national survey. However, the overall mealtimes in this study were 15–20 min later than those reported in the national survey. The difference in breakfast timing between weekdays and weekends was smaller than the national average. (Appendix A, Table A3). Energy intake among participants did not differ significantly from the national average, considering that the new edition of the Standard Tables of Food Composition in Japan used in this study generally yields lower energy estimates than the previous edition used in the 2019 National Health and Nutrition Survey (Appendix A, Table A4). Physical activity levels were generally higher than the national average, except among younger men (Appendix A, Table A5). Sleep duration was slightly longer than the national average (Appendix A, Table A6). Overall, participants in this study were characterized by a notably lower rate of breakfast skipping compared with the general population. Minor differences included slightly higher physical activity levels among women and slightly longer sleep durations among both men and women.

### 3.2. Visualization of Daily Mealtime Trends

#### 3.2.1. Line Plots of Individual Breakfast Timing Trends

**Figure 2:**
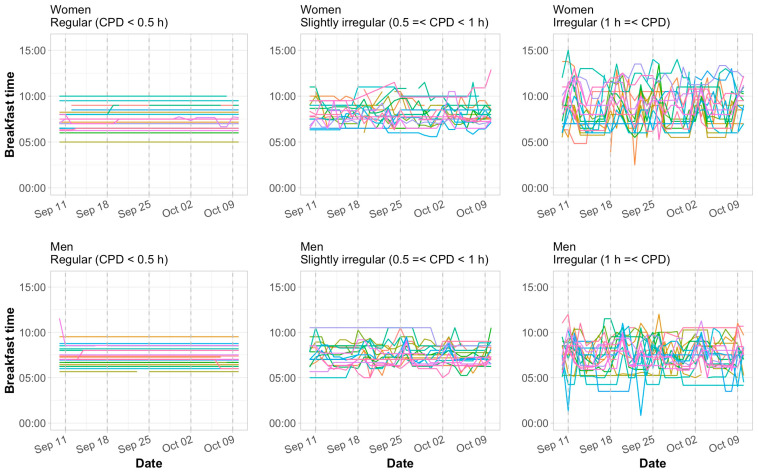
Daily trends in breakfast timing across irregularity groups. Each individual corresponds to a line of a different color. Saturdays are indicated by gray dashed vertical lines. CPD, composite phase deviation.

Figure 2 illustrates the daily changes in breakfast time for representative individuals (each color-coded) from 10 September to 10 October 2021. The left panel shows 20 individuals in the regular group (breakfast CPD < 0.5 h), the right panel shows the irregular group (breakfast CPD ≥ 1 h), and the center panel shows the slightly irregular group (0.5 ≤ breakfast CPD < 1 h) (top row: women; bottom row: men).

No distinct periodicity was observed in breakfast timing. Mealtime variability was not limited to weekends; among individuals in the irregular breakfast group (CPD ≥ 1 h), fewer than 40% exhibited a weekday–weekend difference in breakfast time of 1 h or more (i.e., eating jetlag ≥ 1 h).

#### 3.2.2. Proportional Symbol Plots of Individual Mealtime and Energy Intake Patterns

**Figure 3:**
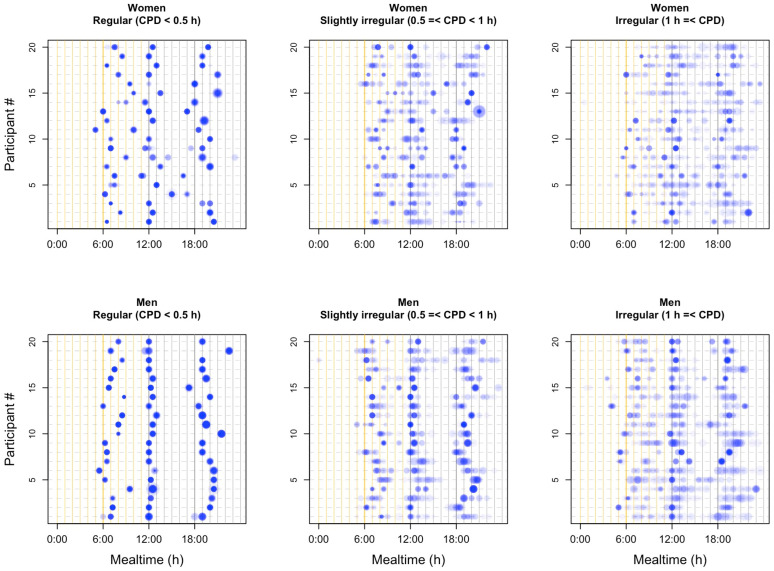
Energy intake and mealtime patterns of individuals. The horizontal axis represents time from 00:00 to 24:00, while each individual is displayed at a distinct vertical position. Each meal is represented by a circle, with color intensity increasing in proportion to the frequency of intake at the same time. The area of each circle is approximately proportional to the corresponding energy intake. CPD, composite phase deviation.

Proportional symbol plots were created to illustrate mealtime patterns during the study period (Figure 3). The left, center, and right panels show representative examples of 20 individuals from the regular, slightly irregular, and irregular breakfast groups, respectively. In the regular group, energy intake occurred at consistent times throughout the day, whereas in the irregular group, meal timing was largely unscheduled. When breakfast timing was irregular, lunch and dinner times also tended to be irregular. This pattern was supported by the strong correlations observed among breakfast, lunch, and dinner CPD values (Table S1).

### 3.3. Chronotype, Sleep–Wake Timing, Mealtime, and Energy Intake Variability Across Mealtime Irregularity Groups

Overall, approximately 70% of both men and women maintained regular mealtimes (CPD < 0.5 h) (Table 2a,b). Figure S1 presents scatter plots illustrating the relationship between mealtime irregularity (CPD) and chronotype (MSFsc). Although no clear proportional relationship was observed between MSFsc and CPD, individuals with extremely high MSFsc values tended to exhibit greater mealtime irregularity across all meals. Figure S2 shows the relationship between average mealtime and CPD. During typical mealtime periods (breakfast: 6:00–8:30; lunch: 12:00–13:00; dinner: 18:00–20:00), later meal timing was generally associated with higher CPD values.

The relationship between breakfast time irregularity and physical, as well as chrono-nutritional, parameters was investigated across age tertiles (Table 2a,b). Among women in the oldest tertile, irregular breakfast timing was associated with several factors: age decreased with irregularity (*p* = 0.0076), BMI increased (*p* = 0.0050), and participants with more irregular breakfast times exhibited a stronger evening chronotype (*p* = 0.0007). Irregularity in breakfast timing was not associated with total daily energy intake but was associated with greater day-to-day variability (CV) in energy intake across all age groups. Irregular breakfast patterns were not related to sleep duration or physical activity. Notably, associations between irregular eating patterns and BMI or other parameters in the upper age tertile of women were observed only for breakfast time irregularity, not for lunch or dinner time irregularity (Tables S2 and S3).

In men, irregular breakfast patterns were not associated with chronotype, sleep–wake rhythms, or energy intake. However, in the younger group, individuals with irregular breakfast patterns tended to have later lunch times (Table 2b).

### 3.4. Association Between BMI and Breakfast Irregularity in Older Women

Multivariate regression analysis was conducted to examine the effect of breakfast time irregularity on BMI (Table 3a,b). After adjusting for age, physical activity level, energy intake, and chronotype—factors known to influence BMI—a positive association between irregular breakfast patterns and BMI was observed in the upper age tertile of women (estimate = 0.740, *p* = 0.0393) (Table 3a). In contrast, among women in the lower age tertile, BMI was associated with age and chronotype (estimate = 1.345, *p* = 0.0302; estimate = 0.861, *p* = 0.0065) (Table 3a). Sensitivity analysis confirmed that the association between BMI and breakfast irregularity was specific to older women (Appendix A, Table A7), showing a significant association in those aged 50 years and older (estimate = 1.098, *p* = 0.0100). No association was observed between irregular eating patterns and BMI in men in our study (Table 3b).

## 4. Discussion

This study is the first to quantitatively evaluate mealtime irregularity and its health effects using nearly daily dietary records collected over approximately 1 month from Japanese adults. Obtaining such long-term, high-frequency dietary data has traditionally been challenging; however, our study overcame this limitation by utilizing data from a food-logging mobile application, enabling a detailed quantitative assessment of mealtime regularity. In the field of chrono-nutrition, evening chronotypes and skipping breakfast have previously been associated with higher BMI [1,2,8,9]. As our inclusion criteria required daily dietary records, only a few participants skipped breakfast. Therefore, this study focused on the relationship between breakfast time irregularity and BMI, adjusting for potential confounding factors, including chronotypes. Our findings revealed a positive association between irregular breakfast timing and BMI among older women. This implies that, although older women tend to have earlier chronotypes (i.e., tend to be “morning-type”), maintaining a consistent, early eating schedule may be important for weight management. In contrast, among younger women, BMI was associated with chronotype, consistent with previous research. Although the specific BMI-related factors varied by age group, the results showed the significant role of habitual mealtime regularity in weight regulation.

Previous studies on irregular eating patterns have employed diverse definitions and assessment methods. Early research used questionnaires to qualitatively evaluate meal regularity, reporting associations with obesity and cardiovascular disease risk. Subsequently, Pot et al. introduced an irregularity score focusing on variability in EI across meals and demonstrated its association with metabolic syndrome [25]. However, most studies have used questionnaires to focus on related concepts such as “eating jetlag,” “breakfast skipping,” or “late-night eating,” which have been linked to obesity and type 2 diabetes [3,4,26,27,28,29]. When mealtime variability was examined, it often referred to differences in mealtimes between workdays and free days, with relatively short observation periods; for instance, eating jetlag has been estimated from 8-day sleep/meal diaries [5] or approximately 7 days of time-stamped dietary data [6]. Consequently, few studies have directly assessed day-to-day variability in actual meal timing.

McHill et al. [21] were the first to apply the CPD metric to quantitatively assess mealtime irregularity, calculating it from 7-day dietary records of university students (*n* = 14). The average CPD time was approximately 3 h, indicating poor day-to-day stability in the timing of caloric intake within individuals. Our study adopted the same calculation method; however, the resulting CPD values were considerably smaller. The smaller CPD values in our study likely reflect differences in the study population, which included both university students and a larger number of working adults with more structured daily routines.

In a large-scale survey of 4032 participants, Murakami et al. reported that most Japanese adults regularly consume three meals per day, rarely skip meals, and snack less frequently than individuals in Western countries [16]. The present study revealed that approximately 18% of participants had CPD values exceeding 1 h, indicating that a subset of Japanese adults exhibits irregular daily mealtimes. Furthermore, individuals with irregular breakfast times tended to have irregular lunch and dinner times, and their mealtimes generally occurred later than the group average.

In this study, we examined the effect of consuming breakfast at irregular times on BMI, adjusting for age, physical activity, EI, and chronotype. The chronotype was strongly associated with BMI in younger women, whereas irregular breakfast timing was associated with BMI in older women. Consistent with previous studies [3,30,31], younger individuals with evening-oriented chronotypes tended to have higher BMI, a pattern replicated in our study. Chronotypes shift toward morning type with age. Among our participants, older women had a significantly earlier mean MSFsc (3.01 ± 1.13) compared to younger women (3.71 ± 1.21). These results suggest that, even as chronotypes shift earlier in older women, irregular breakfast timing may still contribute to weight gain. We speculate that hormonal changes related to menopause and aging may increase sensitivity to irregular meal timing in this group.

While our study focused on BMI, the insights from the recent related studies on normal-weight obesity—a condition not identifiable by BMI alone—suggest broader health implications. The increasing prevalence of normal-weight obesity has become a global health concern and is reported to be associated with unhealthy sleep and dietary habits, such as an evening chronotype, late-night eating, and irregular meal patterns, including breakfast skipping [32,33,34]. These findings are consistent with our results, which showed a significant association between chronotype and BMI, particularly in younger women. However, it is important to note that young Japanese women may have a higher prevalence of pre-sarcopenia than normal-weight obesity [35]. Therefore, further studies are warranted to clarify whether mealtime irregularity is associated with normal-weight obesity or with other health conditions, such as pre-sarcopenia, particularly in the context of young Japanese women.

A noteworthy finding of our study is that the association between mealtime irregularity and BMI was confined to women. One possible explanation is methodological; the smaller sample size for men may have precluded the detection of a statistically significant association. However, this finding also aligns with a growing body of evidence suggesting that women may be more vulnerable to the metabolic consequences of circadian disruption. For instance, stronger associations between late eating and risks for cardiovascular disease [36] and metabolic syndrome [37] have been reported in women than in men. These sex-specific differences may be driven by hormonal factors (e.g., the influence of estrogen on metabolism), as well as sexual dimorphisms in the circadian system [38]. Furthermore, unmeasured lifestyle or social factors, which can differ by sex, may also have contributed to this observation.

The food anticipatory response may underlie the health benefits of regular mealtimes. A well-known example is the pre-meal rise in ghrelin, which develops through habitual, consistent meal timing. This response helps regulate hunger and satiety, prepare the body for upcoming food intake, and facilitate efficient digestion and nutrient absorption, which may help prevent glucose metabolism abnormalities and obesity [39]. Recent studies have confirmed the presence of food anticipatory response in humans [40,41]. Irregular eating patterns can disrupt this system by preventing a clear pre-meal ghrelin surge—a phenomenon observed in animal studies [42,43], which may in turn lead to impaired glucose metabolism. Consistent with this, one study reported that greater variability in the timing of the first daily meal was associated with an increase in HbA1c over 1 year [6]. Although clinical testing was not performed in the present study, future research should investigate the effects of habitual irregular eating habits on glucose metabolism.

A key strength of this study is its use of high-resolution data—daily dietary records over approximately 1 month—to visualize mealtime variability and examine the relationship between breakfast irregularity and BMI. Notably, age-stratified analysis revealed that mealtime regularity remains crucial, even as chronotype shifts toward morning type in older women.

However, our study has several limitations. First, basic participant data, such as age, sex, weight, and height, were self-reported rather than being directly verified by the researchers. Second, potential confounding factors that may influence dietary habits—such as socioeconomic status or living situation—could not be controlled for in the association analyses. Third, reporting bias due to omissions or inaccuracies in self-reported dietary records cannot be ruled out. However, the results are consistent with previous studies that used different mobile app datasets [13], supporting data reliability. Fourth, the study population was limited to Japanese adults, who culturally tend to eat three meals a day regularly. Such cultural characteristics may have introduced selection bias. Nevertheless, comparative analyses between the regular and irregular groups revealed that irregular mealtime was associated with higher BMI. This suggests that regularity of mealtime is an important health factor, even in a population that largely adheres to a three-meal-a-day pattern. Fifth, the study population was limited to mobile application users who consistently recorded their meals. Daily logging requires diligence and health awareness, which may introduce selection bias toward individuals with specific characteristics. Dietary habits, personality, and chronotype are influenced by genetic background [44,45]. Future analyses incorporating these factors are expected to advance personalized chrono-nutrition. Sixth, the dataset did not include information on snack timing, preventing assessment of the association between snacking—particularly late-night eating—and health outcomes. Seventh, an analysis comparing the types of foods consumed by individuals with differing mealtime regularity was not included, but is currently in progress. Finally, the cross-sectional design of this study precludes causal inference. Longitudinal studies, including prospective cohort studies or intervention trials, are needed to determine the causal effects of irregular meal timing on health outcomes.

## 5. Conclusions

Our study used data from a mobile food diary application to quantitatively evaluate and visualize daily mealtime variability in a Japanese population. Greater breakfast time irregularity was positively associated with higher BMI in the older women group. These findings support the notion that maintaining regular eating patterns may help preserve proper physiological responses to anticipated meals, thereby preventing metabolic disorders.

## Figures and Tables

**Table 1:** Participant characteristics.

	Women (*n* = 742)		Men (*n* = 360)	
	Mean	SD	Mean	SD
Age (years old)	41.5	11.1	46.7	11.0
Height (cm)	158.3	5.3	171.6	5.9
Weight (kg)	55.6	10.4	69.5	9.7
BMI (kg/m^2^)	22.2	4.0	23.6	3.0
Wake time (hh:mm)	6:40	1:16	6:17	1:20
Sleep onset time (hh:mm)	23:45	1:11	23:32	1:14
Sleep duration (hh:mm)	6:55	0:58	6:46	0:57
MSFsc (hh:mm)	3:21	1:12	2:55	1:20
SJL (hh:mm)	0:40	0:38	0:30	0:36
Physical activity (MET-h/week)	32.9	30.3	47.4	43.0
Breakfast time (hh:mm)	7:53	1:15	7:24	1:12
Lunch time (hh:mm)	12:39	0:56	12:31	0:45
Dinner time (hh:mm)	19:08	1:12	19:24	1:11
Breakfast time CPD (h)	0.43	0.71	0.42	0.62
Lunch time CPD (h)	0.41	0.65	0.31	0.49
Dinner time CPD (h)	0.37	0.62	0.42	0.66
Rate of breakfast skipping (%)	5.5	13.5	4.5	13.3
Daily energy intake (EI) (kcal/day)	1536	241	2098	345

Abbreviations: BMI, body mass index; MSFsc, sleep-corrected midpoint on free days; SJL, social jetlag; MET, metabolic equivalent of task; CPD, composite phase deviation; EI, energy intake.

**Table 2:** (**a**) Physical and chrono-nutritional measurements in women by age and breakfast time irregularity: mean (upper), (SD) (lower), and *p*-values for trend. (**b**) Physical and chrono-nutritional measurements in men by age and breakfast time irregularity: mean (upper), (SD) (lower), and *p*-values for trend.

(**a**)												
	**Lower Age Tertile** **Age < 36 [*n* = 245]**				**Intermediate Age Tertile #** **36 ≤ Age < 47 [*n* = 240]**				**Upper Age Tertile** **47 ≤ Age [*n* = 257]**			
**Group [*n*]**	**Regular** **[162]**	**Slightly Irregular [31]**	**Irregular [52]**	***p* for Trend**	**Regular** **[161]**	**Slightly Irregular [38]**	**Irregular [40]**	***p* for Trend**	**Regular** **[182]**	**Slightly Irregular [38]**	**Irregular [37]**	***p* for Trend**
Age (years old)	29.2	29.2	28.8	0.3762	40.8	41.0	40.7	0.5737	54.3	53.6	51.9	0.0076 *
	(4.2)	(4.9)	(4.7)		(3.3)	(3.3)	(3.1)		(5.5)	(5.3)	(4.5)	
BMI (kg/m^2^)	21.9	22.8	21.0	0.8281	22.3	23.2	22.0	0.5917	21.9	23.9	22.9	0.0050
	(3.6)	(6.3)	(2.0)		(3.7)	(5.2)	(3.6)		(4.0)	(3.8)	(4.7)	
MSFsc (hh:mm)	3:39	3.26	4:06	0.0787	3:17	3:19	3:32	0.134	2:52	3:11	3:34	0.0007
	(1:10)	(1:14)	(1:17)		(1:07)	(1:13)	(1:16)		(1:03)	(1:07)	(1:21)	
Wake time (hh:mm)	6:59	7:00	7:23	0.1615	6:38	6:39	6:56	0.1276	6:06	6:23	6:59	0.0015
	(1:11)	(1:17)	(1:32)		(1:13)	(1:15)	(1:10)		(1:02)	(1:05)	(1:29)	
Sleep onset time (hh:mm)	23:58	23:44	24:10	0.3427	23:38	23:36	24:03	0.0559	23:23	23:53	24:05	0.0001
	(1:11)	(1:25)	(1:08)		(1:08)	(1:04)	(1:10)		(1:05)	(1:09)	(1:18)	
Sleep duration (h)	7.0	7.3	7.2	0.0621	7.0	7.1	6.9	0.6042	6.7	6.5	6.9	0.2225
	(0.9)	(1.1)	(1.0)		(0.9)	(1.0)	(1.0)		(0.9)	(1.0)	(1.2)	
Breakfast time (hh:mm)	7:56	8:08	8:27	0.0053	7:44	8:04	8:19	<0.0001	7:29	7:48	8:44	<0.0001
	(1:22)	(1:03)	(1:19)		(1:05)	(1:04)	(1:01)		(1:09)	(1:05)	(1:43)	
Lunch time (hh:mm)	12:28	12:49	13:07	<0.0001	12:32	12:46	12:52	0.0007	12:29	12:45	13:23	<0.0001
	(0:51)	(0:37)	(1:11)		(0:46)	(0:39)	(0:58)		(0:55)	(0:41)	(1:25)	
Dinner time (hh:mm)	19:08	19:23	19:25	0.1010	18:59	19:04	19:18	0.0857	19:01	19:04	19:41	0.0039
	(1:17)	(1:37)	(1:23)		(0:55)	(1:01)	(1:22)		(1:11)	(1:00)	(1:14)	
Daily EI (kcal/d)	1508	1512	1486	0.5461	1576	1546	1550	0.8469	1536	1587	1510	0.672
	(240)	(191)	(271)		(254)	(267)	(268)		(196)	(298)	(249)	
Daily EI CV	0.16	0.20	0.24	<0.0001	0.15	0.15	0.18	0.0255	0.13	0.14	0.17	0.0016
	(0.09)	(0.11)	(0.13)		(0.08)	(0.07)	(0.09)		(0.07)	(0.07)	(0.06)	
PA (MET- hour/week)	34.8	37.0	42.9	0.5119	30.0	29.0	29.1	0.411	32.4	28.3	35.9	0.5442
	(31.2)	(29.0)	(50.6)		(26.0)	(29.7)	(16.2)		(28.6)	(21.3)	(34.4)	
(**b**)												
	**Lower Age Tertile** **Age < 42 [*n* = 112]**				**Intermediate Age Tertile #** **42 ≤ Age < 53 [*n* = 128]**				**Upper Age Tertile** **53 ≤ Age [*n* = 120]**			
**Group [*n*]**	**Regular** **[79]**	**Slightly Irregular [15]**	**Irregular [18]**	***p* for Trend**	**Regular** **[77]**	**Slightly Irregular [19]**	**Irregular [31]**	***p* for Trend**	**Regular** **[87]**	**Slightly Irregular [16]**	**Irregular [17]**	***p* for Trend**
Age (years old)	33.4	33.9	33.7	0.3266	46.6	47.1	48.4	0.0056	59.0	57.1	58.6	0.7849
	(5.5)	(6.2)	(5.9)		(3.1)	(3.2)	(3.0)		(4.5)	(3.9)	(3.6)	
BMI (kg/m^2^)	24.0	23.6	23.3	0.7001	23.6	23.3	23.1	0.7173	23.2	24.1	24.3	0.9494
	(3.7)	(3.3)	(2.8)		(3.0)	(2.5)	(2.9)		(2.5)	(3.0)	(2.7)	
MSFsc (hh:mm)	3:32	2:40	4:04	0.3850	2:57	2:56	3:03	0.3687	2:16	2:40	2:15	0.3244
	(1:20)	(1:24)	(1:22)		(1:17)	(1:13)	(1:12)		(1:06)	(0:55)	(1:04)	
Wake time (hh:mm)	6:56	6:21	7:21	0.3755	6:24	6:12	6:16	0.7133	5:35	5:53	5:29	0.5058
	(1:29)	(1:19)	(1:11)		(1:18)	(1:09)	(1:08)		(1:01)	(0:58)	(0:57)	
Sleep onset time (hh:mm)	24:01	23:07	24:22	0.5822	23:37	23:37	23:46	0.2795	22:58	23:26	22:35	0.6141
	(1:12)	(1:22)	(1:24)		(1:14)	(1:08)	(1:03)		(1:04)	(1:00)	(0:50)	
Sleep duration (h)	6.9	7.2	7.0	0.2078	6.8	6.6	6.5	0.8983	6.6	6.5	6.9	0.3948
	(1.1)	(0.7)	(1.0)		(0.9)	(1.0)	(0.8)		(0.9)	(0.8)	(0.7)	
Breakfast time (hh:mm)	7:35	7:38	8:05	0.0308	7:21	7:24	7:42	0.1400	7:04	7:19	7:04	0.1820
	(1:04)	(1:02)	(1:07)		(1:23)	(0:39)	(1:29)		(1:12)	(0:50)	(0:51)	
Lunch time (hh:mm)	12:34	12:33	12:56	0.0020	12:20	12:36	12:48	0.0001	12:27	12:38	12:21	0.1530
	(0:49)	(0:36)	(0:48)		(0:41)	(0:33)	(0:44)		(0:47)	(0:40)	(0:34)	
Dinner time (hh:mm)	19:34	19:32	19:47	0.1665	19:26	19:30	19:41	0.1065	19:08	18:58	18:49	0.7990
	(1:18)	(1:06)	(1:13)		(1:11)	(0:58)	(1:07)		(1:12)	(0:48)	(0:58)	
Daily EI (kcal/d)	2180	2191	2021	0.8523	2083	2063	2116	0.2238	2055	2067	2071	0.3915
	(396)	(300)	(382)		(336)	(368)	(306)		(305)	(280)	(413)	
Daily EI CV	0.15	0.14	0.18	0.2096	0.14	0.17	0.18	0.0074	0.13	0.13	0.18	0.0160
	(0.06)	(0.05)	(0.09)		(0.07)	(0.06)	(0.08)		(0.06)	(0.07)	(0.10)	
PA (METS hour/week)	53.4	39.1	44.9	0.7784	47.8	38.7	48.9	0.6642	47.9	41.6	38.8	0.8110
	(46.6)	(20.7)	(35.5)		(57.9)	(19.7)	(48.6)		(34.9)	(22.9)	(30.8)	

Notes: mean (upper), (SD) (lower), and *p*-values from Jonckheere–Terpstra test (increasing trend; * indicates a decreasing trend) are reported. (a) # One out of 240 individuals in the intermediate group who never ate breakfast was excluded from this analysis. (b) # One out of 128 individuals in the intermediate group who never ate breakfast was excluded from this analysis. Abbreviations: BMI, body mass index; MSFsc, sleep-corrected midpoint on free days; EI, energy intake; PA, physical activity; MET, metabolic equivalent of task.

**Table 3:** (**a**) Multivariate regression analysis of factors associated with body mass index by age tertile in women. (**b**) Multivariate regression analysis of factors associated with body mass index by age tertile in men.

(**a**)									
	**Lower Age Tertile** **Age < 36**			**Intermediate Age Tertile** **36 ≤ Age < 47**			**Upper Age Tertile** **47 ≤ Age**		
**Risk Factor**	**Coefficients**	**95% CI**	***p*-Value**	**Coefficients**	**95% CI**	***p*-Value**	**Coefficients**	**95% CI**	***p*-Value**
Age	1.345	[0.136, 2.553]	0.0302	0.019	[−1.691, 1.728]	0.9830	0.708	[−0.341, 1.756]	0.1869
PA	−0.035	[−0.446, 0.375]	0.8663	−0.31	[−0.911, 0.300]	0.3240	−0.366	[−0.899, 0.166]	0.1789
EI	0.178	[−0.312, 0.668]	0.4772	0.30	[−0.170, 0.775]	0.2110	0.421	[−0.126, 0.968]	0.1330
Chronotype	0.861	[0.246, 1.476]	0.0065	0.53	[−0.107, 1.173]	0.1040	0.478	[−0.176, 1.131]	0.1536
Breakfast time irregularity	−0.373	[−0.956, 0.209]	0.2101	−0.07	[−0.731, 0.583]	0.8260	0.740	[0.040, 1.440]	0.0393
(**b**)									
	**Lower Age Tertile** **Age < 42**			**Intermediate Age Tertile** **42 ≤ Age < 53**			**Upper Age Tertile** **53 ≤ Age**		
**Risk Factor**	**Coefficients**	**95% CI**	***p*-Value**	**Coefficients**	**95% CI**	***p*-Value**	**Coefficients**	**95% CI**	***p*-Value**
Age	0.535	[−0.674, 1.743]	0.4238	−0.788	[−2.578, 1.002]	0.3900	0.824	[−0.397, 2.044]	0.1886
PA	0.689	[0.279, 1.100]	0.0627	0.233	[−0.189, 0.654]	0.2810	−0.622	[−1.241, 0.004]	0.0511
EI	−0.091	[−0.581, 0.399]	0.7789	−0.352	[−0.873, 0.170]	0.1890	0.194	[−0.323, 0.711]	0.4631
Chronotype	−0.046	[−0.661, 0.569]	0.9065	0.488	[−0.125, 1.101]	0.1210	0.386	[−0.309, 1.080]	0.2783
Breakfast time irregularity	−0.322	[−0.904, 0.261]	0.4703	−0.179	[−0.783, 0.426]	0.5640	0.505	[−0.131, 1.140]	0.1225

Notes: Age, PA, and EI were standardized using z-score transformation. Chronotype was coded as follows: 0 = morning type; 1 = intermediate type; and 2 = evening type. Breakfast time irregularity was coded as 0 = regular, 1 = slightly irregular, and 2 = irregular. Abbreviations: CI, confidence interval; PA, physical activity; EI, energy intake. Abbreviations: CI, confidence interval; PA, physical activity; EI, energy intake.

## Data Availability

The data used in this study are the property of the company and will not be released to the public due to privacy reasons. However, the data will be provided from the company to researchers, upon request, for research purposes.

## Supplementary Materials

The following supporting information can be downloaded a https://www.mdpi.com/article/10.3390/nu17223504/s1. Figure S1. Scatter plot showing the relationship between MSFsc and composite phase deviation for each meal; Figure S2. Scatter plot showing the relationship between mealtime and composite phase deviation for each meal; Table S1. Spearman’s rank correlation among breakfast, lunch, and dinner time irregularity metrics, composite phase deviation (CPD); Table S2. Physical and chrono-nutritional measurements in women by age and lunch-time irregularity. Table S3. Physical and chrono-nutritional measurements in women by age and dinner-time irregularity.

## Abbreviations

The following abbreviations are used in this manuscript:

BMI	Body mass index
CPD	Composite phase deviation
Hb	Hemoglobin
MET	Metabolic Equivalent of Task
EI	Energy intake
CV	Coefficient of variation
MSFsc	Corrected sleep midpoint on free days
PA	Physical activity
CI	Confidence interval

## Appendix A

**Table A1:** BMI characteristics of participants by sex and age in the present study, compared with data from the National Health and Nutrition Survey, Japan [46].

	Mean BMI [kg/m^2^] (*n*)			
	Women		Men	
	The Present Study	NHNS Japan in 2019	The Present Study	NHNS Japan in 2019
age < 30	21.5 (116)	20.7 (216)	23.6 (27)	22.2 (226)
30 ≤ age < 40	22.2 (227)	21.7 (214)	23.9 (69)	23.7 (177)
40 ≤ age < 50	22.4 (199)	22.3 (356)	23.5 (110)	24.7 (295)
50 ≤ age < 60	22.3 (161)	22.4 (377)	23.3 (105)	24.6 (286)
60 ≤ age	22.6 (39)	23 (1235)	23.9 (49)	23.7 (1064)

Notes: Mean BMI (*n*) is reported. Abbreviations: BMI, body mass index; NHNS, National Health and Nutrition Survey.

**Table A2:** Proportion of participants skipping breakfast by sex and age in this study, compared with the 2023 Awareness Survey on Food Education, Ministry of Agriculture, Forestry and Fisheries (MAFF) [47].

Breakfast		Women		Men	
		The Present Study	MAFF Survey in 2023	The Present Study	MAFF Survey in 2023
20 ≤ age < 40		*n* = 336	*n* = 267	*n* = 95	*n* = 167
	almost every day (%)	91.4	64.0	92.6	58.1
	4–5 days a week (%)	5.4	10.1	5.3	8.4
	2–3 days a week (%)	2.1	9.7	2.1	9.6
	rarely eat (%)	1.2	16.1	0.0	22.8
	no response (%)	0.0	0.0	0.0	1.2
40 ≤ age < 60		*n* = 360	*n* = 425	*n* = 215	*n* = 348
	almost every day (%)	95.0	77.4	93.0	71.0
	4–5 days a week (%)	3.1	6.6	3.3	4.9
	2–3 days a week (%)	1.7	6.6	2.3	4.9
	rarely eat (%)	0.3	8.9	1.4	18.7
	no response (%)	0.0	0.5	0.0	0.6
60 ≤ age		*n* = 39	*n* = 607	*n* = 49	*n* = 495
	almost every day (%)	89.7	89.0	100.0	86.9
	4–5 days a week (%)	5.1	2.5	0.0	4.2
	2–3 days a week (%)	2.6	2.6	0.0	2.2
	rarely eat (%)	2.6	4.0	0.0	5.3
	no response (%)	0.0	2.0	0.0	1.4

Notes: Participants in this study, aligned with the MAFF survey, were classified as follows: almost every day: consuming breakfast on ≥79% of recorded days; 4–5 days: consuming breakfast on 50–78% of recordeddays; 2–3 days: consuming breakfast on 21–49% of recorded days; rarely eat: consuming breakfast on <21% of recorded days. Abbreviations: MAFF, Ministry of Agriculture, Forestry and Fisheries.

**Table A3:** Sex-stratified average breakfast and dinner times on weekdays, Saturdays, and Sundays in this study, compared with the 2021 Survey on Time Use and Leisure Activities (Statistics Bureau of Japan) [48].

	Women		Men	
	The Present Study	2021 Survey (Statistics Bureau of Japan)	The Present Study	2021 Survey (Statistics Bureau of Japan)
Breakfast (Weekdays)	7:50	7:18	7:20	7:05
Dinner (Weekdays)	19:08	18:49	19:25	19:07
Breakfast (Saturday)	8:01	7:36	7:33	7:28
Dinner (Saturday)	19:05	18:37	19:18	18:44
Breakfast (Sunday)	7:58	7:46	7:29	7:40
Dinner (Sunday)	19:07	18:33	19:21	18:40

**Table A4:** Daily energy intake (kcal/d) by sex and age in this study, compared with data from the National Health and Nutrition Survey, Japan [46].

	Women		Men	
	The Present Study	NHNS 2019	The Present Study	NHNS 2019
20 ≤ age < 30	1492	1600	2234	2199
30 ≤ age < 40	1543	1673	2156	2081
40 ≤ age < 50	1561	1729	2093	2172
50 ≤ age < 60	1539	1695	2081	2188
60 ≤ age < 70	1546	1784	1992	2177

Notes: Energy intake in this study was calculated using the 8th edition of the Standard Tables of Food Composition, whereas the National Health and Nutrition Survey 2019 used the 7th edition. Abbreviations: NHNS, National Health and Nutrition Survey.

**Table A5:** Physical activity levels (MET-h/week) by sex and age in this study, compared with data from the Sasagawa Sports Foundation National Survey, Japan [49].

	Women		Men	
	The Present Study	National Survey 2020	The Present Study	National Survey 2020
age < 30	37.7 (116)	29.8 (215)	41.4 (27)	64.7 (227)
30 ≤ age < 40	33.9 (227)	24.1 (219)	50.8 (69)	60.7 (234)
40 ≤ age < 50	29.2 (199)	23.7 (287)	50.4 (110)	51.6 (299)
50 ≤ age < 60	32.5 (161)	24.7 (248)	46.7 (105)	39.8 (249)
60 ≤ age	33 (39)	18.3 (521)	40.6 (49)	31.4 (468)

Notes: Mean MET-h per week (*n*) is reported.

**Table A6:** Distribution of sleep duration by sex in this study, compared with data from the National Health and Nutrition Survey, Japan [46].

	Women		Men	
	The Present Study % (*n*)	NHNS 2019 % (*n*)	The Present Study % (*n*)	NHNS 2019 % (*n*)
Sleep duration (h) < 5	2.2 (16)	9.1 (276)	3.1 (11)	8.5 (227)
5 ≤ Sleep duration (h) < 6	11.6 (86)	31.5 (955)	13.3 (48)	29 (773)
6 ≤ Sleep duration (h) < 7	35.0 (260)	36.2 (1098)	37.8 (136)	32.7 (873)
7 ≤ Sleep duration (h) < 8	37.1 (275)	16.8 (510)	36.7 (132)	20.1 (536)
8 ≤ Sleep duration (h) < 9	11.2 (83)	4.8 (145)	6.9 (25)	7.1 (190)
9 ≤ Sleep duration (h)	3.0 (22)	1.6 (49)	2.2 (8)	2.6 (69)

**Table A7:** Sensitivity analysis using alternative age stratification: multivariate regression of risk factors for body mass index in women.

	Age < 35 (*n* = 224)			35 ≤ Age < 50 (*n* = 318)			50 ≤ Age (*n* = 200)		
Risk Factor	Coefficients	95% CI	*p*-Value	Coefficients	95% CI	*p*-Value	Coefficients	95% CI	*p*-Value
Age	1.943	[0.58, 3.3]	0.006	0.324	[−0.77, 1.41]	0.560	0.797	[−0.53, 2.12]	0.240
PA	−0.020	[−0.45, 0.41]	0.926	−0.328	[−0.84, 0.19]	0.212	−0.398	[−0.98, 0.19]	0.184
EI	0.120	[−0.39, 0.63]	0.646	0.350	[−0.07, 0.77]	0.101	0.435	[−0.19, 1.06]	0.174
Chronotype	0.852	[0.2, 1.51]	0.011	0.588	[0.05, 1.13]	0.033	0.378	[−0.38, 1.14]	0.332
Breakfast time irregularity	−0.399	[−1.03, 0.23]	0.216	−0.090	[−0.63, 0.45]	0.744	1.098	[0.27, 1.93]	0.010

Notes: Age, PA, and EI were scaled using z-score transformation. Chronotype was coded as follows: 0 = morning type, 1 = intermediate type, 2 = evening type. Breakfast time irregularity was coded as follows: 0 = regular, 1 = slightly irregular, 2 = irregular. Abbreviations: CI, confidence interval; PA, physical activity; EI, energy intake.
