# Supplementary material for: Day-to-Day Variability in Meal Timing and Its Association with Body Mass Index: A Study Using Data from a Japanese Food-Logging Mobile Application
Source: Nutrients. 2025 Nov 9;17(22):3504. doi: 10.3390/nu17223504 (PMC12655051; doi:10.3390/nu17223504)
Supplement: Supplementary file 1 [file nutrients-17-03504-s001.zip › Supplementary_information.pdf]

Figure S1. Scatter plot showing the relationship between MSFsc and composite phase deviation for each meal

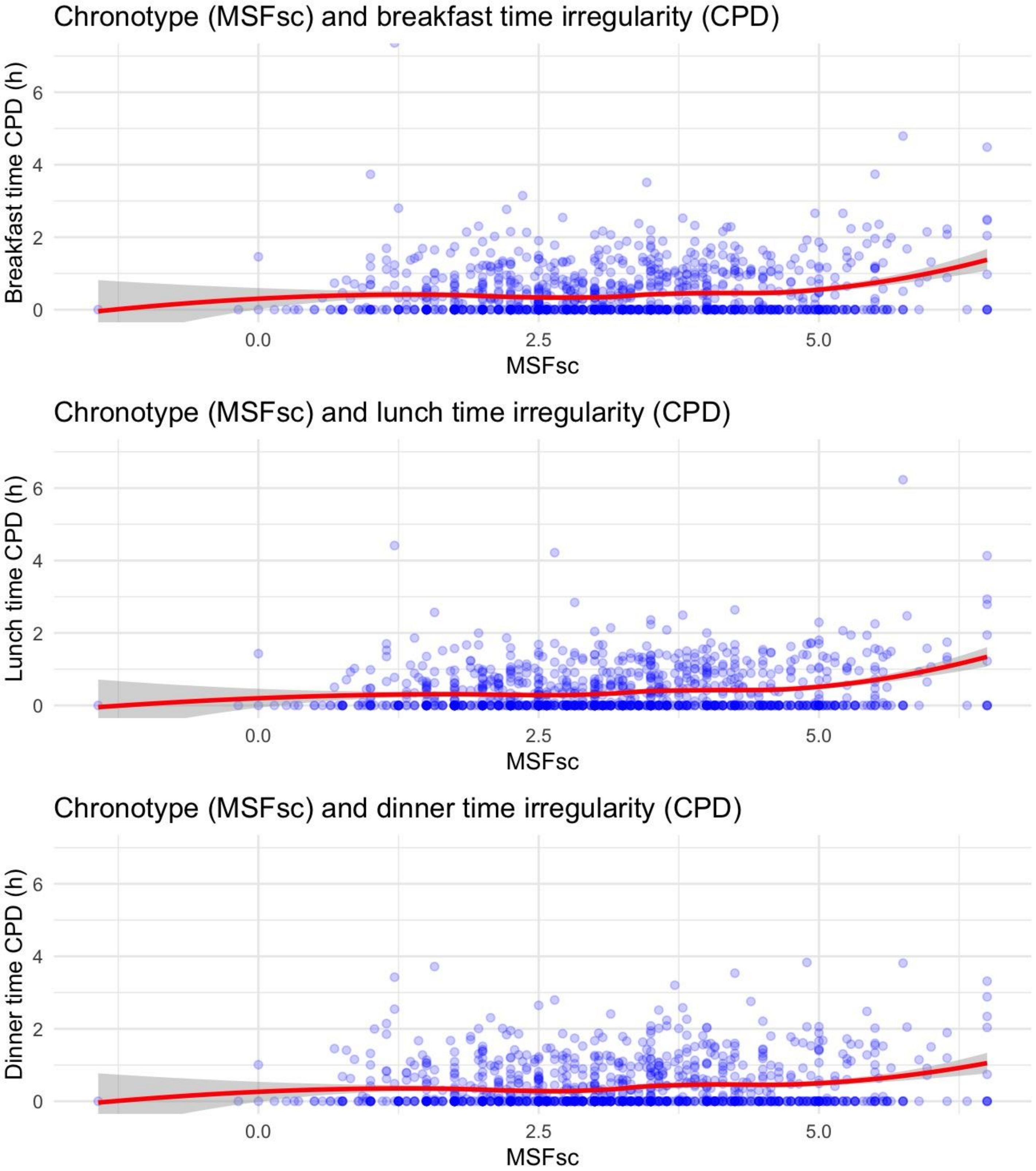

In this study, chronotypes were classified into morning (MSFsc < 2.57), intermediate (2.57 ≤ MSFsc < 3.75), and evening (3.75 ≤ MSFsc) types based on the MSFsc tertiles. The regression curve (red) was drawn using the LOESS (locally estimated scattered smoothing) method. Gray areas indicate 95% confidence intervals. MSFsc, Sleep-corrected midpoint on free days; CPD, composite phase deviation.

Figure S2. Scatter plot showing the relationship between mealtime and composite phase deviation for each meal.

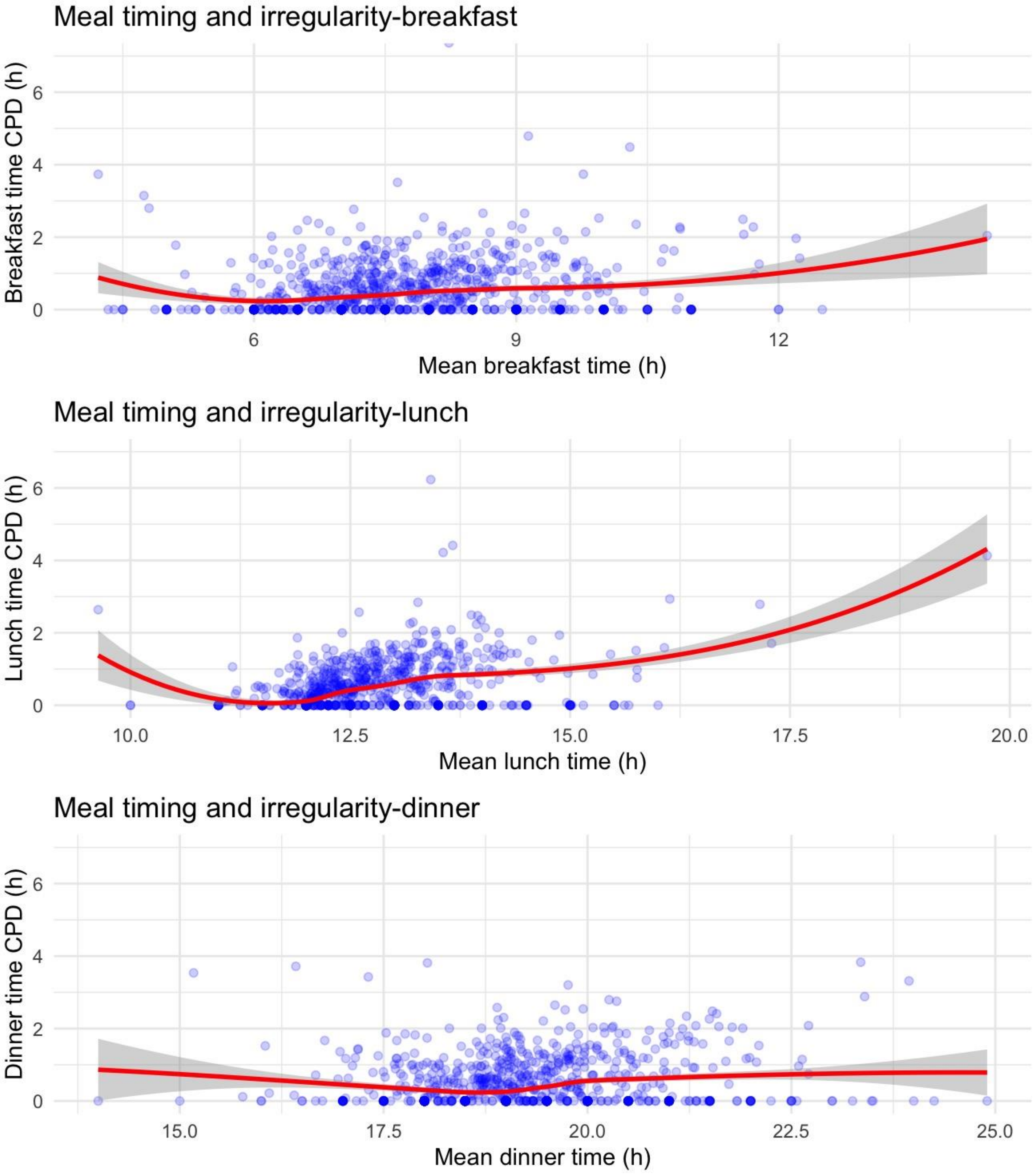

The regression curve (red) was drawn using the LOESS (locally estimated scattered smoothing) method. Gray areas indicate 95% confidence intervals. CPD, composite phase deviation.

Table S1. Spearman's rank correlation among breakfast, lunch, and dinner time irregularity metrics, measured by composite phase deviation

a. Women

	Breakfast time CPD	
Lunch time CPD	0.738 ($p < 2.2 \times 10^{-16}$)	Lunch time CPD
Dinner time CPD	0.720 ($p < 2.2 \times 10^{-16}$)	0.778 ($p < 2.2 \times 10^{-16}$)

b. Men

	Breakfast time CPD	
Lunch time CPD	0.808 ($p < 2.2 \times 10^{-16}$)	Lunch time CPD
Dinner time CPD	0.775 ($p < 2.2 \times 10^{-16}$)	0.779 ($p < 2.2 \times 10^{-16}$)

CPD, composite phase deviation.

Table S2. Physical, chrono-nutritional measurements in women by age and lunch-time irregularity.

	**Lower age tertile**				**Intermediate age tertile**				**Upper age tertile**			
	Age < 36 (n = 245)				36 ≤ age < 47 (n = 240)				47 ≤ age (n = 257)			
Group (n)	Regular	Slightly irregular	Irregular	*p* for Trend	Regular	Slightly irregular	Irregular	*p* for Trend	Regular	Slightly irregular	Irregular	*p* for Trend
	(n = 166)	(n = 36)	(n = 43)		(n = 158)	(n = 44)	(n = 38)		(n = 181)	(n = 31)	(n = 45)	
Age (yrs old)	29.3	29.3	28.2	0.1972	40.6	41.3	41.1	0.9040	54.3	54.1	51.9	0.0039*
	4.1	5	4.8		3.3	3	3.2		5.4	6.3	4.4	
BMI (kg/m^2)	22	21.9	21	0.9506	22.2	22.2	23.7	0.0719	22.1	23.8	22.3	0.1133
	4	4.1	2.8		3.7	3.8	4.8		4.1	4.2	4.2	
MSFsc (hh:mm)	3:36	3:36	4:06	0.0574	3:18	3:06	3:48	0.0842	2:48	3:18	3:36	< 0.0001
	1:12	1:12	1:24		1:12	1:00	1:18		1:00	1:18	1:12	
Wake time (hh:mm)	6:56	6:51	7:43	0.0177	6:39	6:27	7:11	0.0751	6:06	6:23	6:59	0.0001
	1:10	1:07	1:38		1:15	0:59	1:16		1:02	1:05	1:29	
Bedtime (hh:mm)	23:55	23:57	24:16	0.0945	23:37	23:38	24:12	0.0209	23:23	23:53	24:05	< 0.0001
	1:12	1:03	1:20		1:08	1:02	1:09		1:05	1:09	1:18	
Sleep duration (h)	7	6.9	7.5	0.0209	7	6.8	7	0.8097	6.7	7	6.5	0.4654
	0.9	0.9	1.1		0.9	1	1.1		1	0.9	1.1	
Breakfast time (hh:mm)	7:53	8:09	8:40	0.0005	7:45	7:55	8:26	< 0.0001	7:26	8:11	8:27	< 0.0001
	1:20	1:07	1:21		1:06	0:49	1:11		1:13	1:08	1:25	
Lunch time (hh:mm)	12:25	12:52	13:22	< 0.0001	12:25	12:51	13:13	< 0.0001	12:26	12:57	13:23	< 0.0001
	0:49	0:48	1:07		0:41	0:41	0:59		0:53	0:50	1:17	
Dinner time (hh:mm)	19:11	19:13	19:26	0.0968	18:54	19:07	19:42	0.0007	19:00	19:13	19:33	0.0014
	1:22	1:02	1:31		0:50	0:55	1:32		1:08	1:08	1:22	
Daily EI (kcal/d)	1505	1501	1503	0.3984	1572	1532	1583	0.7291	1546	1460	1569	0.8785
	230	319	209		256	213	307		196	240	291	
Daily EI CV	0.16	0.2	0.24	< 0.0001	0.14	0.15	0.19	0.0092	0.13	0.14	0.16	0.0005
	0.09	0.09	0.15		0.08	0.08	0.09		0.07	0.06	0.06	
PA (MET-hour/week)	36.2	38	37.9	0.7407	29.1	30.5	30.4	0.1597	32.2	29.1	34.6	0.2447
	33.9	43.2	37.7		26.3	19.4	27		29.2	28.3	26.2	

Notes. mean (upper), SD (lower) and *p*-values from Jonckheere–Terpstra test (increasing trend; * indicates a decreasing trend) are reported. Abbreviations: BMI, body mass index; MSFsc, sleep-corrected midpoint on free days; EI, energy intake; PA, physical activity; MET, metabolic equivalent of task.

Table S3. Physical, chrono-nutritional measurements in women by age and dinner-time irregularity.

	Lower age tertile				Intermediate age tertile				Upper age tertile#			
	Age < 36 (n = 245)				36 ≤ age < 47 (n = 240)				47 ≤ age (n = 257)			
Group (n)	Regular	Slightly irregular	Irregular	*p* for Trend	Regular	Slightly irregular	Irregular	*p* for Trend	Regular	Slightly irregular	Irregular	*p* for Trend
	(n = 170)	(n = 31)	(n = 44)		(n = 174)	(n =33)	(n = 33)		(n = 190)	(n = 29)	(n = 37)	
Age (yrs old)	29.2	29.4	28.6	0.4035	40.9	39.8	41.3	0.3189	54.4	52.2	52.4	0.0044*
	4.3	4.9	4.7		3.3	2.6	3.3		5.6	3.7	4.8	
BMI (kg/m^2)	21.9	22.3	21.2	0.8267	22.3	23.6	22	0.3731	22.1	23.4	22.3	0.1796
	4	4	3		3.7	4.8	4		4.1	4.5	4.1	
MSFsc	3:40	3:35	4:01	0.1606	3:17	3:30	3:32	0.0883	2:53	3:13	3:31	0.0026
	1:11	1:08	1:19		1:08	1:16	1:10		1:03	1:08	1:24	
Wake time (hh:mm)	6:58	6:53	7:35	0.0587	6:37	6:51	6:59	0.0244	6:07	6:30	6:51	0.0056
	1:12	1:06	1:35		1:13	1:22	1:04		1:01	1:08	1:34	
Bedtime (hh:mm)	23:56	23:55	24:12	0.2247	23:37	23:46	24:09	0.0140	23:26	23:45	24:03	0.0036
	1:12	1:15	1:15		1:08	1:03	1:09		1:07	1:11	1:17	
Sleep duration (h)	7	7	7.4	0.0157	7	7.1	6.8	0.7261	6.7	6.7	6.8	0.3218
	0.9	1.2	1		0.9	1	1		0.9	0.9	1.4	
Breakfast time (hh:mm)	7:54	8:05	8:43	0.0006	7:47	8:08	8:14	< 0.0001	7:30	7:54	8:37	< 0.0001
	1:18	1:09	1:23		1:05	1:05	1:06		1:12	1:11	1:34	
Lunch time (hh:mm)	12:31	12:45	13:06	< 0.0001	12:30	12:49	13:05	< 0.0001	12:31	12:43	13:19	< 0.0001
	0:51	0:48	1:13		0:43	0:37	1:05		0:55	0:39	1:28	
Dinner time (hh:mm)	19:10	19:05	19:33	0.0597	18:57	19:09	19:35	0.0042	19:04	18:59	19:26	0.0908
	1:19	0:55	1:40		0:53	1:06	1:27		1:09	1:06	1:24	
Daily EI (kcal/d)	1517	1407	1522	0.7648	1576	1546	1550	0.8063	1538	1543	1537	0.7754
	239	299	183		254	267	268		190	232	341	
Daily EI CV	0.16	0.19	0.23	< 0.0001	0.15	0.15	0.18	0.0189	0.13	0.13	0.18	< 0.0001
	0.1	0.09	0.13		0.08	0.07	0.09		0.07	0.06	0.06	
PA (MET-hour/week)	38.1	31.9	35	0.9235	30	29	29.1	0.3923	32.6	34.5	28.7	0.6105
	37	31.5	35		26	29.7	16.2		28.9	35.8	19.9	

Notes. mean (upper), SD (lower), and *p*-values from Jonckheere–Terpstra test (increasing trend; * indicates a decreasing trend) are reported. #One out of 257 individuals in the upper age group who never ate dinner was excluded from this analysis. Abbreviations: BMI, body mass index; MSFsc, sleep-corrected midpoint on free days; EI, energy intake; PA, physical activity; MET, metabolic equivalent of task.
